# Supplementary material for: Induced Resistance Combined with RNA Interference Attenuates the Counteradaptation of the Western Flower Thrips
Source: Int J Mol Sci. 2022 Sep 17;23(18):10886. doi: 10.3390/ijms231810886 (PMC9500759; doi:10.3390/ijms231810886)
Supplement: Supplementary file 1 [file ijms-23-10886-s001.zip › ijms-1926131-supplementary/Table S5_primer.pdf]

Table 1. Primers for RT-qPCR and dsRNA synthesis.

Gene	Primer use	Sequence (5′–3′)	Product length (bp)
FoGSTs1	qPCR	F： CTAGCCAAGCTGGTCGGATT<br>R： GGGTCGTAGTACCATTCGCT	116
FoGSTd1	qPCR	F： TGCACCAGAGGCTGTACTTC<br>R： TTCTTCACCTTCTCCGCGTC	109
FoGSTt1	qPCR	F： TCAACCATCACGGGCATTGA<br>R： CTGGAACTCGTCCAAACCGA	134
FoGSTe1	qPCR	F： TCAACCCACTGCACACAGTT<br>R： CTGGTTCACAATGGCCCTCT	146
dsFoGSTs1	dsRNA synthesis	F：<br>TAATACGACTCACTATAGGGACAAGATTTTTGCCCA<br>ATCG<br>R：<br>TAATACGACTCACTATAGGGTCGATTCCACCTTCTT<br>GACC	487
	qPCR	F： GCACCCAAATACGTACTCCA<br>R： TCCTCCCAGTTGATATTGCC	92
dsFoGSTd1	dsRNA synthesis	F： TAATACGACTCACTATAGGG<br>CGACTTCTACATGATGCCC<br>R：<br>TAATACGACTCACTATAGGGGTAGTAGATCTCCCCG<br>AAAC	337
	qPCR	F： CTTCCTGAGCGAGTCCAAGT<br>R： ACCTGACGATGTTGGGGAAC	128
eGFP	dsRNA synthesis	F： TAATACGACTCACTATAGGG<br>CCCTGAAGTTCATCTGCACC<br>R：<br>TAATACGACTCACTATAGGGGTGCTCAGGTAGTGGT<br>TGTC	481
EF-1	qPCR	F: TCAAGGAACTGCGTCGTGGAT<br>R: ACAGGGGTGTAGCCGTTAGAG	130
